# Glucagon-Like Peptide-1 Receptor Agonist Treatment Does Not Reduce Abuse-Related Effects of Opioid Drugs

**DOI:** 10.1523/ENEURO.0443-18.2019

**Published:** 2019-04-24

**Authors:** Annika Billefeld Bornebusch, Anders Fink-Jensen, Gitta Wörtwein, Randy J. Seeley, Morgane Thomsen

**Affiliations:** 1 Laboratory of Neuropsychiatry, Psychiatric Centre Copenhagen, Rigshospitalet, Mental Health Services, Capital Region of Denmark, Copenhagen 2100, Denmark and University Hospital of Copenhagen, Copenhagen DK2100, Denmark; 2 Department of Clinical Medicine, Faculty of Health and Medical Sciences, University of Copenhagen, Copenhagen 2100, Denmark; 3 Department of Surgery, University of Michigan, Ann Arbor, MI 48109

**Keywords:** abuse, gut-brain axis, neuropeptides, operant behavior, opioid, substance use disorder

## Abstract

Dependence on opioids and the number of opioid overdose deaths are serious and escalating public health problems, but medication-assisted treatments for opioid addiction remain inadequate for many patients. Glucagon-like pepide-1 (GLP-1) is a gut hormone and neuropeptide with actions in peripheral tissues and in the brain, including regulation of blood glucose and food intake. GLP-1 analogs, which are approved diabetes medications, can reduce the reinforcing and rewarding effects of alcohol, cocaine, amphetamine, and nicotine in rodents. Investigations on effects of GLP-1 analogs on opioid reward and reinforcement have not been reported. We assessed the effects of the GLP-1 receptor agonist Exendin-4 (Ex4) on opioid-related behaviors in male mice, i.e., morphine-conditioned place preference (CPP), intravenous self-administration (IVSA) of the short-acting synthetic opioid remifentanil, naltrexone-precipitated morphine withdrawal, morphine analgesia (male and female mice), and locomotor activity. Ex4 treatment had no effect on morphine-induced CPP, withdrawal, or hyperlocomotion. Ex4 failed to decrease remifentanil self-administration, if anything reinforcing effects of remifentanil appeared increased in Ex4-treated mice relative to saline. Ex4 did not significantly affect analgesia. In contrast, Ex4 dose dependently decreased oral alcohol self-administration, and suppressed spontaneous locomotor activity. Taken together, Ex4 did not attenuate the addiction-related behavioral effects of opioids, indicating that GLP-1 analogs would not be useful medications in the treatment of opioid addiction. This difference between opioids and other drug classes investigated to date may shed light on the mechanism of action of GLP-1 receptor treatment in the addictive effects of alcohol, central stimulants, and nicotine.

## Significance Statement

Opioid overdoses are now the leading cause of death for age under 50 in the United States, and effective new treatments to curb opioid addiction worldwide are urgently needed. Glucagon-like pepide-1 (GLP-1) analogs are safe, approved, diabetes medications that have shown promising “anti-addiction” effects in preclinical studies with alcohol, central stimulants, and nicotine. In the present study, we report that GLP-1 receptor stimulation does not attenuate rewarding or reinforcing effects of opioid drugs and does not attenuate acquisition or expression of opioid withdrawal.

## Introduction

Opioid dependence is a still rapidly increasing public health problem (Hedegaard et al., 2017, Seth et al., 2018). Death by drug overdose has become the leading cause of death for age under 50 in the United States (data are from the Multiple Cause of Death Files, 1999–2016, as compiled from data provided by the 57 vital statistics jurisdictions through the Vital Statistics Cooperative Program; Centers for Disease Control and Prevention, National Center for Health Statistics, 2017). The current treatment options for opioid addiction are limited and they require continuous daily monitoring by health professionals to be safe and effective (Volkow et al., 2014; Volkow and Collins, 2017). There is therefore an urgent need to address the ongoing and worsening opioid epidemic and identify better medication-assisted treatments. Glucagon-like peptide-1 (GLP-1) is a polypeptide with both hormone and neuropeptide functions. It is produced in the intestinal system, where it is released in response to nutrient ingestion, as well as in the brain (Holst, 2013). GLP-1 decreases food intake and body weight, and has homeostatic effects on blood glucose by activating insulin secretion and decreasing glucagon secretion (Drucker et al., 2017). This combination of effects makes GLP-1 receptor agonists like Exendin-4 (Ex4; exenatide) an effective treatment for type 2 diabetes, an indication for which they have been used for over a decade (Eng et al., 2014; Andersen et al., 2018).

GLP-1 receptors are expressed in peripheral tissues like the pancreas, and in many brain regions, and GLP-1 regulates feeding behavior and food intake at least in part through central mechanisms (Skibicka, 2013; Sisley et al., 2014; van Bloemendaal et al., 2014). GLP-1 receptors are also found in areas associated with reward, reinforcement, and addiction including the ventral tegmental area and nucleus accumbens (Göke et al., 1995; Merchenthaler et al., 1999; Alhadeff et al., 2012; Cork et al., 2015; Heppner et al., 2015). In humans, gene variants of the GLP-1 receptor are associated with increased prevalence of alcohol use disorder and with increased responses to alcohol in laboratory studies (Suchankova et al., 2015). Diabetes patients treated with the GLP-1 analog liraglutide reported decreases in alcohol intake (Kalra et al., 2011). In rodents and non-human primates, systemic or central administration of Ex4 and other GLP-1 receptor agonists attenuate addiction-related effects of alcohol, cocaine, amphetamine, and nicotine, including self-administration/drinking, conditioned place preference (CPP), and striatal dopamine efflux (Graham et al., 2013; Egecioglu et al., 2013a,b,c; Shirazi et al., 2013; Suchankova et al., 2015; Sørensen et al., 2015, 2016; Reddy et al., 2016; Vallöf et al., 2016; Fortin and Roitman, 2017; Tuesta et al., 2017; Thomsen et al., 2017, 2018). Those findings indicate that GLP-1 receptor stimulation modulates the rewarding and reinforcing effects of drugs of abuse and that GLP-1 receptor agonists represent a possible treatment for addictions (Fink-Jensen and Vilsbøll, 2016). At present, Ex4 is being evaluated in clinical trials for the management of alcohol use disorder and for smoking cessation, respectively (Antonsen et al., 2018; Yammine et al., 2018). Despite these promising findings and the fact that GLP-1 receptor agonists are already approved as diabetes medications, making them easily available for treatment of drugs and alcohol use disorders, the possible effects of GLP-1 receptor agonists on addiction-related effects of opioids have not yet been reported.

Here, we tested GLP-1 receptor stimulation using Ex4 on three abuse-related effects of opioid drugs: (1) rewarding effects, using a CPP assay with morphine; (2) the direct reinforcing effects, using intravenous self-administration (IVSA) of the fast-onset short-duration opioid agonist remifentanil (Panlilio and Schindler, 2000); (3) dependence/withdrawal, using antagonist-precipitated morphine withdrawal. The IVSA experiment included transgenic knock-out (KO) mice lacking GLP-1 receptors in the central nervous system selectively, following the hypothesis that Ex4 would decrease remifentanil IVSA in WT mice, but not in the KO mice. A secondary hypothesis was that remifentanil IVSA would be increased in the KO mice, because whole-body GLP-1 receptor KO mice were reported to self-administer more nicotine and have stronger cocaine-induced CPP than WT mice (Harasta et al., 2015; Tuesta et al., 2017). Furthermore, because of the clinical importance of opioids in pain management, we investigated whether Ex4 treatment would interfere with the therapeutic properties of morphine, an important consideration if GLP-1 receptor agonists were to be used in the treatment of addiction to opioids. For this we used a hot-plate assay, which is thought to involve brain processing, rather than a spinal reflex (Le Bars et al., 2001). Finally, we verified the effectiveness of Ex4 using endpoints previously reported to be sensitive to GLP-1 receptor stimulation, namely locomotor activity and oral alcohol self-administration, measured in direct comparison to the opioid assays.

## Materials and Methods

### Animals

*Glp1r* ^flox/flox^ nestin-Cre^+/−^ KO mice with a neuronal-specific deletion of the GLP-1 receptor was generated as described by Sisley et al. (2014) and bred in the animal facility at the Panum Institute, University of Copenhagen. The *Glp1r* ^flox/flox^ nestin-Cre^-/-^ littermates were used as wild type (WT) controls, genotype was determined by polymerase chain reaction on DNA extracted from ear clip samples. Male C57BL/6NTac (B6) mice were acquired at seven to eight weeks of age (Taconic, Denmark). All mice were acclimated to the housing facilities at Laboratory of Neuropsychiatry (AAALAC accredited) at least 7 d before the experiments were initiated. Mice were kept on a reverse 12/12 h light/dark cycle under temperature and humidity control, and all experiments were performed in the dark phase. Tap water and regular rodent chow were available ad libitum, mice were group housed up to eight per cage with hiding devices, nesting material, and wooden chewing block as enrichment. Procedures were approved by the Animal Experiments Inspectorate under the Danish Ministry of Food, Agriculture, and Fisheries in accordance with the European Union directive 2010/63/EU.

### Apparatus

Behavioral studies were conducted in equipment from Med Associates: mouse modular operant-conditioning chambers (ENV-307A) for self-administration studies (Thomsen and Caine, 2005; Thomsen et al., 2009), and open field activity chambers (OFA 510) for place conditioning studies (Dall et al., 2017). All chambers were individually enclosed in sound-attenuating cubicles equipped with a white light and a ventilation fan. The operant-conditioning chambers contained two nose-poke holes each fitted with a photocell and a yellow cue light, and a low-torque liquid swivel (375/25; Instech Laboratories) mounted on a balance arm was used for intravenous drug delivery from a syringe pump in the freely moving animals. The activity chambers for CPP were fitted with a beam-break movement detection system. An unbiased two-compartment design was used, with distinct floor materials (metal wire grid vs gray plastic Lego plate) in the two compartments. For the hot-plate assay, a Hot Plate Analgesia Meter (Harvard Apparatus) was used at 55 ± 0.2°C, with a floor consisting of a 10-mm aluminum plate, 20 cm in diameter, enclosed by a clear plastic cylinder. In the withdrawal assay clear plastic cylinders ∼20 cm in diameter and 60 cm tall were used for observation.

### CPP and locomotor activity

Male B6 mice were used. Mice were habituated to the test room for at least 60 min before the beginning of the session. During pre-conditioning (day 1) and post-conditioning test (day 10) mice were allowed to move freely between the two compartments for 30 min. No individual mice showed preference on the pre-conditioning day as defined by ≥75% time spent on one side, and mice showed no significant preference as a group. During conditioning sessions (days 2–9), mice were confined to the Lego or grid compartment for 40 min. The mice were assigned to treatment in counterbalanced groups after the pre-conditioning day based on their time spent in the two compartments and balanced for chamber, side, floor texture and treatment sequence. During conditioning sessions, mice were injected with Ex4 (10 μg/kg), then, 30 min later, with morphine (10 mg/kg) and immediately placed in the designated compartment. On the alternating days, the mice received saline injections and were placed in the non-drug paired compartment, with a total of eight once-daily pairings. Control groups received saline injections paired with both compartments, and either Ex4 (10 μg/kg) or saline pretreatment. This resulted in four treatment groups: saline + saline (Sal+Sal), saline + morphine (Sal+Mor), Ex4 + saline (Ex4+Sal), and Ex4 + morphine (Ex4+Mor). No injections were given before the pre-conditioning or post-conditioning test sessions. Distance traveled for locomotor activity and time spent in each compartment was recorded for all sessions.

### Operant behaviors

Male *Glp1r* ^flox/flox^ nestin-Cre^+/−^ KO and WT mice were used. To test the efficacy of Ex4 treatment in reducing opioid self-administration behavior under conditions that favor acquisition of drug taking, mice were allowed to acquire nose-poking reinforced with a palatable liquid food (vanilla flavor Nutridrink), followed by extinction, before catheter implantation. In addition, to test for general differences in operant performance between genotypes, food-reinforced behavior was examined further in a subset of mice.

#### Food-reinforced operant behaviors

Mice were mildly food restricted starting 6 d before and until completion of the food-reinforced studies, to no less than 85% of their free-feeding weight. The liquid food was first presented in the home cage (5 ml in a plastic cup) 3 d before initiating operant access. Mice were then allowed to respond under a fixed ratio (FR) 1 timeout 20-s schedule of food reinforcement 5 d/week, as previously described (Thomsen et al., 2005) with a few modifications: session time was shortened to 60 min, and minimum reinforcers earned to meet criteria were adjusted to 15/session, and the concentration series was tested only once. In brief, testing comprised: acquisition (minimum five sessions and until criteria were met, which was never more than nine sessions); extinction to <50% of acquisition reinforcers (minimum three sessions, all met criteria within three sessions); concentration-response curve (0%, 3%, 10%, 32%, 100% food in water presented in a Latin square design). Then, undiluted food was again available for one session, followed by two consecutive sessions under a logit-based progressive ratio (PR) schedule as previously described (Thomsen et al., 2005). Finally, nose-poking behavior was again extinguished in at least three sessions under the FR 1 schedule before catheter implantation. Additional mice used for remifentanil self-administration studies were exposed to the acquisition and extinction phases of the food training only, with the same criteria.

#### Catheter implantation and maintenance

The methods of the catheter manufacturing, surgical implantation, and maintenance have been described in detail (Thomsen and Caine, 2005; Stoll et al., 2018). In brief, the catheters consisted of medical-grade silicone tubing (1-French size; Phymep) attached to a 90°-angled cannula (Bilaney) fixed in a dental cement base with a 20-mm disk of polypropylene surgical mesh (Textile Development Associates). Under sevoflurane anesthesia the catheter was implanted in the jugular vein and ran subcutaneously to the catheter base located above the midscapular region. The mice were allowed a 7-d recovery period with daily infusions of 0.03-ml saline containing heparin (30 USP units/ml) and antibiotics (67-mg/ml cefazolin). The external end of the catheter was kept sealed outside of self-administration sessions. Catheter patency was verified by loss of muscle tone and clear signs of anesthesia within 3 s after the infusion of 0.02- to 0.03-ml ketamine/midazolam through the catheter (15-mg/ml ketamine, 0.75-mg/ml midazolam in saline). Data were only included when catheter patency was confirmed before and after completion of an experimental phase.

#### Remifentanil self-administration

For the self-administration experiment, we chose to administer Ex4 or saline daily throughout testing, for two reasons: first, acute effects of candidate medications often fail to translate to the more clinically relevant effects of repeated/chronic treatment (Haney and Spealman, 2008; Czoty et al., 2016), and second, based on the lack of effect of Ex4 in other assays, we decided to test for effects on initial remifentanil self-administration. If the acute reinforcing effects of remifentanil could not be attenuated, it seemed even less likely that well-established drug taking could be modulated by Ex4.

General self-administration procedures have been described in detail (Thomsen and Caine, 2005; Thomsen et al., 2005), remifentanil doses were based on previous studies in mice (Caine et al., 2007). Mice were randomly assigned to either saline or 10-µg/kg Ex4 before starting testing, treatments were administered 30 min before each session. Mice were allowed to self-administer remifentanil under an FR 1 timeout 20-s schedule of reinforcement, 5–6 d/week. The dose was first varied individually every 3–4 d based on our experience that this can facilitate acquisition (Caine et al., 2014), 10- or 32-µg/kg/infusion. Sessions lasted 3 h or until the maximum allowed reinforcers were earned, 100 at 10 µg/kg and 30 at 32 µg/kg. Mice were considered to self-administer when they took at least 15 reinforcers per session for two consecutive sessions with >75% responses in the active hole, on both doses. Saline was then substituted for remifentanil until responding decreased to <50% of acquisition reinforcers, followed by at least one session on the 10-µg/kg dose and until responding increased to the pre-extinction level or re-stabilized at >15 reinforcers per session, which occurred in all mice that maintained catheter patency. Response requirement was then increased to FR 3, FR 5, and PR in successive sessions. Finally, self-administration of 32-µg/kg/infusion remifentanil and of saline were examined under the PR schedule. Doses were repeated under the PR schedule when catheters were patent long enough, the first and second determinations did not differ significantly and were averaged for analysis.

#### Ethanol self-administration experiment

Male B6 mice were used. Mice were first exposed to ethanol (20% in water w/v) using a drinking in the dark procedure (Rhodes et al., 2005), replacing the home cage water bottles with ethanol solution for 4 h/d for 4 d, starting 3 h after onset of the dark cycle. Then, ethanol solution was made available using the same procedure as the food-reinforced operant responding described above except that sessions lasted 2 h. After three weeks, mice that maintained an average consumption of at least 2.5-g/kg/session ethanol over the last week were considered “drinkers” and were tested in a counterbalanced sequence with saline, 3.2-, and 10-µg/kg Ex4, administered 30 min before the session. Test sessions were separated by at least one baseline session to observe ethanol intake at pre-testing levels. This experiment was performed concurrently with remifentanil studies and using the same batch of Ex4.

### Antagonist-precipitated morphine withdrawal

Mice from the CPP experiment plus experimentally naive B6 mice were used. Mice from the CPP experiment were allowed a 7-d washout period before progressing to the withdrawal experiment, in which they received the same treatment as previously (i.e., morphine-exposed mice remained in a morphine group, etc.). Morphine dependence was induced by 6 d of twice-daily morphine injections of increasing dose, in the home cage (from 10 to 50 mg/kg). On the test day mice received a last morphine dose (50 mg/kg) and 2-h later withdrawal symptoms were precipitated with naltrexone (0.32 mg/kg). Separate cohorts of mice were used to test the effect of Ex4 (3.2 or 10 μg/kg) administered on the test day 30 min before naltrexone (testing for effect on expression of withdrawal), or of repeated Ex4 (10 μg/kg) 30 min before each morphine dose (testing for effect on induction of dependence). Immediately after the naltrexone injection, the mice were placed individually in clear plastic cylinders and behavior was video recorded for 30 min, then mice were killed. The video was analyzed and the number of vertical jumps per 30 min was recorded by an observer blind to treatment condition.

### Hot-plate assay for antinociceptive effect of morphine

Male and female *Glp1r* ^flox/flox^ nestin-Cre^+/−^ KO and WT mice were used (42 WT males, 51 WT females, 52 KO males, and 56 KO females, equally distributed between drug dose groups). Data analysis showed no difference between the female and male groups; therefore, the data are presented as sexes combined. Mice were administered Ex4 (10 μg/kg), followed after 60 min by morphine (5 or 10 mg/kg), and after a further 60 min, the mice were placed on the hotplate. Nociceptive time latency, defined as the time required to elicit a hind paw lick or a jump, was recorded and the animal was removed from the plate. To minimize the risk of tissue damage, mice were removed if no response was recorded within 40 s, recording time as 40 s, and mice were killed immediately after the experiment.

### Drugs

Morphine hydrochloride, remifentanil hydrochloride (B. Braun), ketamine hydrochloride (Pfizer), midazolam hydrochloride (Matrix Pharmaceuticals), sevoflurane (AbbVie), cefazoline (MIP Pharma), and heparin (preservative-free, SAD) were purchased from the Copenhagen University Hospital Pharmacy. Ex4 was purchased from Tocris Bioscience, and naltrexone hydrochloride was purchased from Sigma-Aldrich (product N3136). Alcohol (ethyl alcohol 96% undenatured) was purchased from Plum A/S. Doses refer to the weight of the salts. All drugs were dissolved in sterile saline (0.9% NaCl), alcohol was diluted in tap water. All drugs were administered intraperitoneally in a volume of 10 ml/kg, except for the twice-daily morphine withdrawal regimen, which was administered subcutaneously.

### Experimental design and statistical analysis

#### CPP

Time on drug-paired side in pretest and posttest was analyzed with two-tailed paired-sample *t* test with Bonferroni correction. The % change in time spent in the drug-paired compartment between pre-test and posttest (100× posttest/pretest) was analyzed by ANOVA with treatment as between-subject factor, followed by Bonferroni posttest. Locomotor activity: distance moved was analyzed by ANOVA with genotype and Ex4 treatment as between-subjects factors and consecutive session as repeated-measures factor. Operant food: reinforcers earned per session were analyzed by ANOVA with genotype as between-subjects factor and consecutive session number or food concentration as repeated-measures factor. Operant remifentanil: infusions earned per session were analyzed by ANOVA with genotype and Ex4 dose as between-subjects factor and consecutive session number, or remifentanil dose, or response requirement as repeated-measures factor. Operant ethanol: g/kg earned per session was analyzed by ANOVA with Ex4 dose as repeated-measures factor, followed by Bonferroni posttest. Withdrawal: jumps per 30 min. were analyzed by ANOVA with Ex4 treatment, morphine treatment, and naltrexone treatment as between-subject factors. In mice dependent on morphine and challenged with naltrexone, jumps were analyzed by ANOVA with Ex4 dose as between-subject factor. Significant effects were followed by simple effects test (ANOVA) where applicable, then Bonferroni posttest.

#### Hot-plate

Due to the cutoff value, hot-plate latency data were not normally distributed, nor could they readily be transformed into normality, precluding the use of ANOVA. Latency-to-event data are most appropriately analyzed by survival statistics (Jahn-Eimermacher et al., 2011). Time to response was analyzed by Cox proportional hazard regression with gene, sex, and Ex4 dose as factors; then re-analyzed with sexes combined as the sex factor never approached significance. Data had to be analyzed separately for each morphine dose to not violate the proportionality assumption.

A priori power analyses were performed using G*power 2 (Faul et al., 2007) and Stata. For CPP, analysis was based on the effect sizes reported for acquisition of CPP using alcohol (Egecioglu et al., 2013c; Vallöf et al., 2016), both analyses yielding a required *n* = 9. For withdrawal and hotplate no data on GLP-1 analog effects were available, and a conservative estimate was based on an effect size of 25% reduction, yielding a required of *n* = 11–12. For self-administration, the primary hypothesis was that Ex4 would decrease self-administration of remifentanil in WT mice under an FR 1 schedule of reinforcement, therefore analysis was based on the effect size observed for a relatively high dose of self-administered cocaine (Sørensen et al., 2015) or nicotine (Tuesta et al., 2017) under FR schedules: both calculations yielded a required *n* = 6. Data were analyzed using GraphPad Prism or Stata, significance level was set at *p* < 0.05. All data are presented as group means ± SEM.

## Results

### CPP and locomotor activity

#### Place conditioning

**Figure 1.**
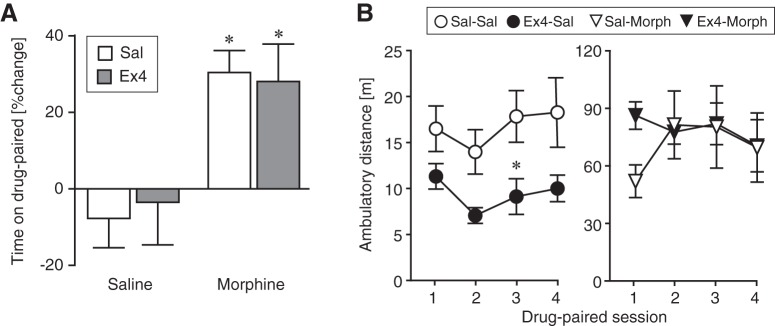
CPP. ***A***, CPP is shown as % increase in time spent in the drug-paired chamber (100× posttest/pretest). Morphine-conditioned mice (10 mg/kg) showed significant place preference compared to the saline-saline control group whether they were pretreated with saline or Ex4 (10 μg/kg). There was no significant effect of Ex4 either alone (saline-conditioned mice) or in morphine-conditioned-mice. ***B***, Distance moved per session in meters as a function of successive drug conditioning sessions; note the different ordinate scaling for saline-conditioned and morphine-conditioned groups; *p < 0.05 versus saline-saline. Data are group mean ± SEM; *n* = 9–10.*Figure Contributions*: Annika Billefeld Bornebusch collected the data. Morgane Thomsen designed the experiments. Annika Billefeld Bornebusch and Morgane Thomsen analyzed the data.

CPP, measured as percentage increase in time spent in the drug-paired compartment (Fig. 1*A*), was increased by morphine conditioning relative to saline (*F*_(1,35)_ = 15.7, *p* = 0.0004, ANOVA, Bonferroni correction), with no effect of Ex4 treatment. The time spent on drug-paired side in the pretest and posttest was also analyzed for each treatment group and morphine-conditioning produced a significant place preference in both saline-pretreated mice and Ex4-pretreated mice (*t*_(9)_ = 4.99, *p* = 0.0008, paired-sample *t* test and *t*_(8)_ = 3.26, *p* = 0.012, paired-sample *t* test, respectively), while saline conditioning had no effect on place preference.

#### Locomotor activity

Locomotor activity as distance moved per session was also recorded during all sessions in the CPP experiment. Figure 1*B* shows distance moved during the conditioning sessions. Morphine increased locomotor activity markedly (*F*_(1102)_ = 51.5, *p* < 0.0001, main effect, three-way ANOVA), with a significant morphine by Ex4 interaction (*F*_(1102)_ = 5.53, *p* = 0.02, three-way ANOVA). Ex4 treatment significantly reduced distance moved in the saline-conditioned groups (*F*_(1,52)_ = 7.40, *p* = 0.01, two-way ANOVA followed by Bonferroni posttest), but not did not significantly modulate morphine-induced locomotor hyperactivity (*p* = 0.7). Groups did not differ in distance moved during pre- or post-conditioning test sessions; similarly, during non-drug conditioning sessions distance moved remained at 10–20 m/session in all groups (data not shown).

### Operant behaviors

#### Food-reinforced operant behaviors

**Figure 2.**
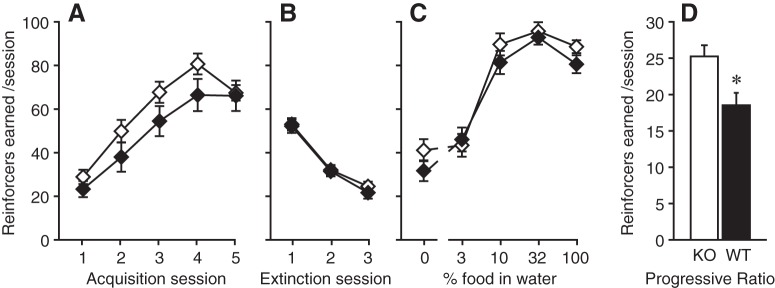
Food-reinforced operant behaviors. Reinforcers earned per session under an FR 1 schedule of reinforcement, as a function of (***A***) successive acquisition sessions, (***B***) successive extinction sessions, (***C***) reinforcer magnitude (dilutions of liquid food in water), and (***D***) with undiluted food under a PR schedule of reinforcement. KO mice (open symbols) earned significantly more reinforcers under the PR schedule but were comparable to WT mice (black symbols) in all other phases of testing; *p < 0.05 versus KO; *n* = 18–21.*Figure Contributions*: Annika Billefeld Bornebusch and Saiy Kiasari collected the data. Morgane Thomsen designed the experiments. Annika Billefeld Bornebusch and Morgane Thomsen analyzed the data.

Male GLP-1 receptor nestin-Cre KO and WT mice were allowed to acquire nose-poking reinforced with liquid food (Fig. 2*A*). Food was then replaced with water (Fig. 2*B*), then a series of food reinforcer dilutions were presented (Fig. 2*C*). Finally, undiluted food was made available under a PR schedule of reinforcement for two sessions (shown averaged, Fig. 2*D*). The two genotypes did not differ significantly in any behaviors under the FR 1 schedule of reinforcement: reinforcers earned increased as a function of number of acquisition sessions (*F*_(4148)_ = 59.1, *p* < 0.0001) and decreased as a function of extinction sessions (*F*_(2,73)_ = 148.1, *p* < 0.0001), and reinforcers earned varied as a function of food concentration (*F*_(4148)_ = 111.7, *p* < 0.0001), with no effect of genotype or interactions with genotype (all main effect, two-way ANOVA). When tested under an increased response requirement, the KO mice reached higher breaking points (more reinforcers earned) than the WT mice (*F*_(1,37)_ = 7.01, *p* = 0.01, one-way ANOVA).

#### Remifentanil self-administration

**Figure 3.**
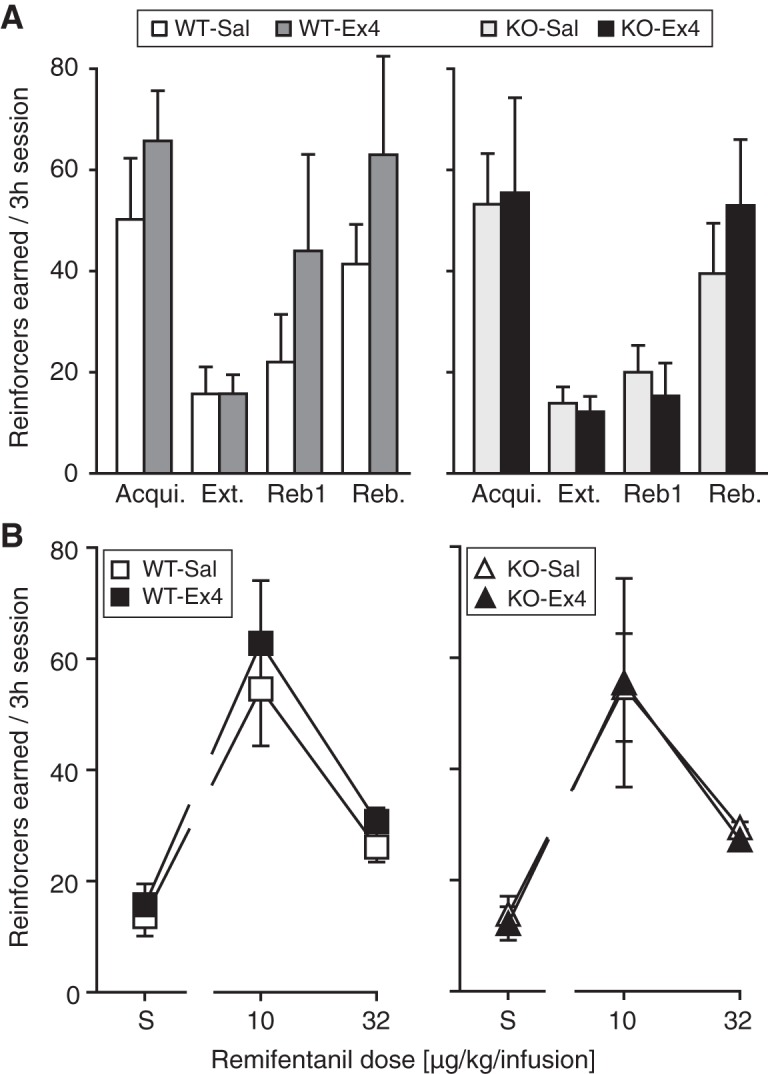
Remifentanil IVSA under an FR 1 schedule of reinforcement. Reinforcers earned per 3-h session in saline-treated and Ex4-treated WT mice and in saline-treated and Ex4-treated KO mice. ***A***, Reinforcers earned at acquisition criteria (Acqui., 10-µg/kg/infusion remifentanil), at extinction criteria (Ext., saline), on the first day of post-extinction self-administration rebaseline (Reb1) and at rebaseline criteria (Reb.). Ex4 treatment appeared to increase remifentanil self-administration in the WT mice, supported by a significant genotype by treatment interaction. ***B***, Reinforcers earned as a function of remifentanil unit dose (µg/kg/infusion); reinforcers were significantly related to dose but not to genotype or Ex4 treatment. KO, *n* = 5–7; WT, *n* = 6–7.*Figure Contributions*: Annika Billefeld Bornebusch and Saiy Kiasari collected the data. Morgane Thomsen designed the experiments. Annika Billefeld Bornebusch and Morgane Thomsen analyzed the data.

Saline-treated or Ex4-treated (10 µg/kg) KO and WT groups did not differ in the rate of acquisition or number of remifentanil reinforcers earned under acquisition. However, there was a tendency for Ex4-treated WT mice to increase responding to acquisition level following extinction (“rebaseline”) more rapidly and to a higher intake relative to saline-treated WT mice. This observation was supported statistically by a significant genotype by treatment interaction (*F*_(1,47)_ = 6.99, *p* = 0.01, three-way ANOVA) on remifentanil reinforcers (or saline infusions) earned per session at acquisition criteria, extinction criteria, and on the first and last (= criteria) session of rebaseline (Fig. 3*A*), although *post hoc* comparisons did not reach significance. Reinforcers earned were also related to training phase (*F*_(3,47)_ = 24.4, *p* < 0.0001, main effect, three-way ANOVA), with no other significant interactions. Self-administration behavior was related to remifentanil dose under the FR 1 schedule of reinforcement (*F*_(2,46)_ = 45.7, *p* < 0.0001, main effect, three-way ANOVA) with highest intake at 10 µg/kg/infusion remifentanil, regardless of treatment and genotype (overall Bonferroni posttest vs saline, 10-µg/kg/infusion, *p* < 0.001, 32-µg/kg/infusion, *p* = 0.007; Fig. 3*B*).

**Figure 4.**
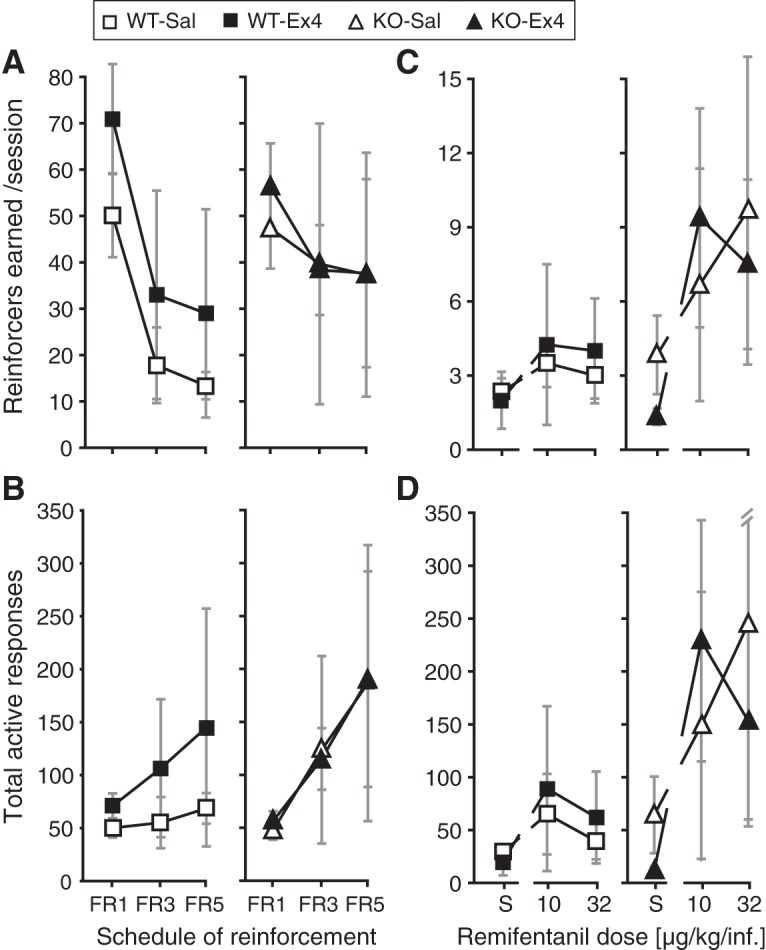
Remifentanil IVSA under increased response requirement. Reinforcers earned (***A***, ***C***) and total active responses (***B***, ***D***) per session in saline-treated and Ex4-treated WT mice and in saline-treated and Ex4-treated KO mice. ***A***, ***B***, With 10-µg/kg/infusion remifentanil available as the reinforcer, response requirement was increased successively from FR 1 to FR 3, FR 5 schedule of reinforcement, resulting in decreasing remifentanil intake in all groups. ***C***, ***D***, Under a PR schedule of reinforcement, saline (S), 10- and 32-µg/kg/infusion remifentanil were made available. In ***A***, ***B***, KO, *n* = 3; WT, *n* = 4–5; in ***C***, ***D***, KO, *n* = 3–4; WT, *n* = 4–6.*Figure Contributions*: Annika Billefeld Bornebusch and Saiy Kiasari collected the data. Morgane Thomsen designed the experiments. Annika Billefeld Bornebusch and Morgane Thomsen analyzed the data.

When the response requirement to earn infusions of 10 µg/kg/infusion remifentanil was increased in successive sessions in a smaller number of mice, behavior was marked by great variability due to one to two mice in all groups defending their intake (i.e., >20 reinforcers earned under the FR 5 schedule) but most mice decreasing intake steeply. Overall, reinforcers earned decreased as a function of cost (*F*_(2,20)_ = 5.64, *p* = 0.01, main effect, three-way ANOVA; FR 3, *p* = 0.050 Bonferroni posttest vs FR 1; FR 5, *p* = 0.02 vs FR 1; Fig. 4*A*) with no other factors or interactions approaching significance. Although not enough to maintain intake levels, mice did increase active responses as a function of cost (*F*_(2,20)_ = 4.13, *p* = 0.03, main effect, three-way ANOVA; none significant *post hoc*; Fig. 4*B*). Again, the tendency was for Ex4-treated WT mice to earn more remifentanil reinforcers and emit more responses relative to saline-treated WT mice. Finally, 10- and 32-µg/kg/infusion remifentanil and saline were made available under the PR schedule of reinforcement, data were again marked by variability due to the same one to two mice in each group showing high intake. Reinforcers earned (*F*_(2,24)_ = 4.78, *p* = 0.02; Fig. 4*C*) and total active responses (*F*_(2,24)_ = 4.41, *p* = 0.02; Fig. 4*D*) were related to remifentanil dose only (main effects, three-way ANOVA).

#### Ethanol self-administration experiment

**Figure 5.**
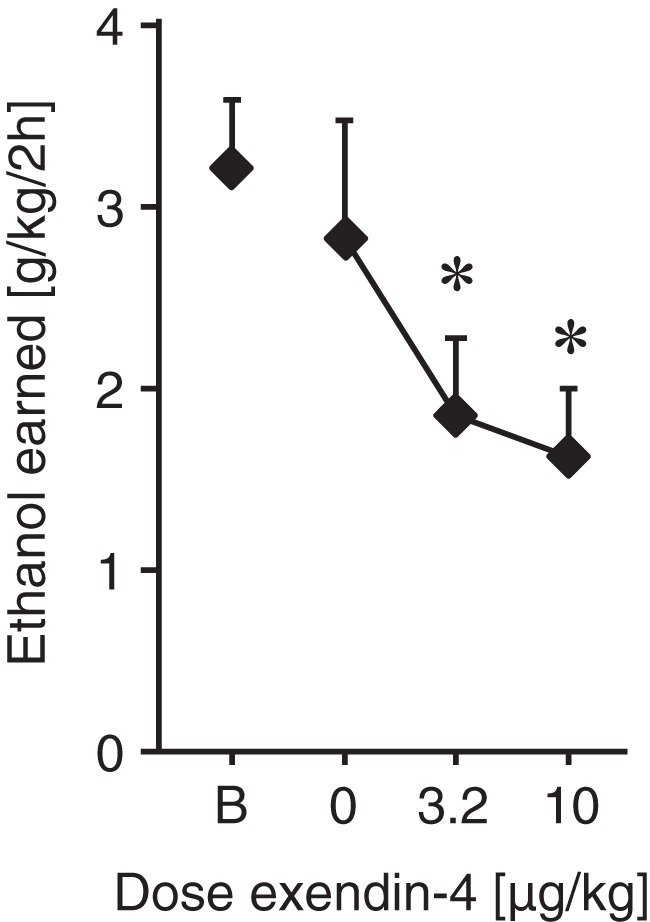
Ethanol self-administration experiment. Ethanol intake (g/kg/2-h session) as a function of Ex4 dose (µg/kg) or baseline intake (B). As a direct comparison, the effect of Ex4 on operant behavior reinforced with oral ethanol solution was assessed, under an FR 1 schedule of reinforcement. Both Ex4 doses decreased ethanol intake significantly; *p* < 0.05 versus baseline; *n* = 7–9.*Figure Contributions*: Annika Billefeld Bornebusch and Saiy Kiasari collected the data. Morgane Thomsen designed the experiments. Annika Billefeld Bornebusch analyzed the data.

Using the same operant conditioning chambers and the same batch of Ex4, concurrently with the remifentanil IVSA studies, we confirmed that Ex4 can decrease responding maintained by oral ethanol under an FR 1 schedule of reinforcement (*F*_(3,18)_ = 5.52, *p* = 0.007, one-way ANOVA; Fig. 5), both at 3.2 µg/kg (*p* = 0.04, Bonferroni posttest vs baseline) and at 10 µg/kg (*p* = 0.02).

### Antagonist-precipitated morphine withdrawal

**Figure 6.**
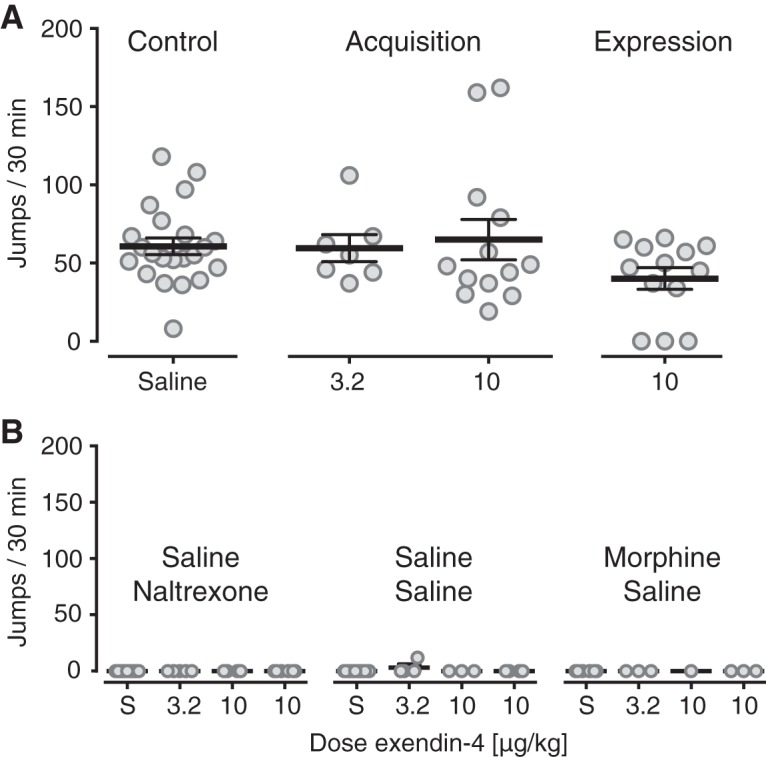
Antagonist-precipitated morphine withdrawal. Somatic withdrawal symptoms measured as jumps per 30 min. ***A***, In mice made dependent with daily morphine injections and challenged with naltrexone (0.32 mg/kg) on the test day, naltrexone precipitated withdrawal symptoms in all groups, with no significant effect of Ex4 administration either as daily pretreatment to morphine (acquisition of morphine dependence; 3.2 and 10 μg/kg) or on the test day (expression of withdrawal; 10 μg/kg), relative to mice treated with saline (control). Control data are shown pooled for mice receiving saline during acquisition or expression. ***B***, Control groups treated with saline and naltrexone, saline-saline, or morphine-saline did not show withdrawal symptoms and showed no effect of Ex4 treatments. Data are group mean ± SEM as well as all individual points; ***A***, *n* = 7–22; ***B***, *n* = 1–14. *Figure Contributions*: Annika Billefeld Bornebusch collected the data. Morgane Thomsen designed the experiments. Annika Billefeld Bornebusch and Morgane Thomsen analyzed the data.

The somatic symptoms of morphine withdrawal were measured as jumps per 30 min. and are shown in Figure 6, the control data for mice receiving saline during acquisition and expression are shown pooled. There was a significant interaction of morphine and naltrexone treatment (*F*_(1,87)_ = 34.2, *p* < 0.001, three-way ANOVA), inducing morphine withdrawal and jumping. In the morphine-dependent groups treated with naltrexone there was no significant effect of Ex4 treatments (*p* = 0.2, one-way ANOVA; Fig. 6*A*). There was also no significant effect of Ex4 treatment in the non-withdrawal control groups (Fig. 6*B*).

### Antinociceptive effect of morphine

**Figure 7.**
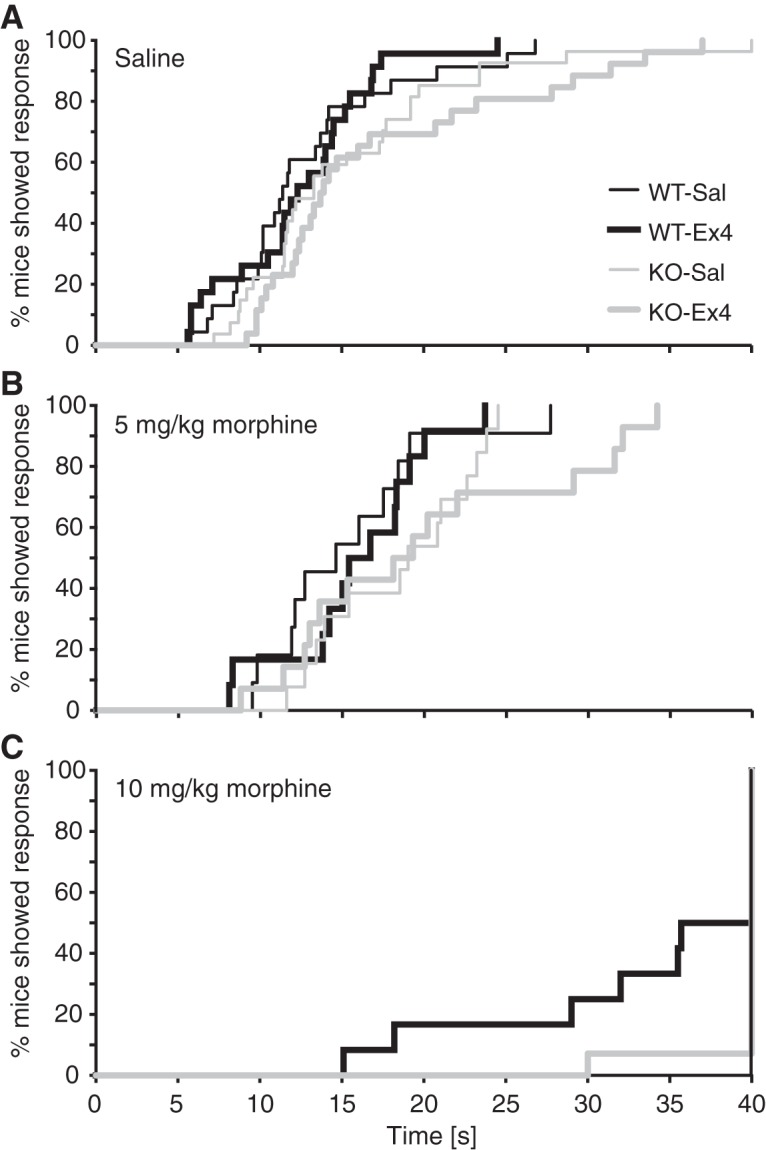
Antinociceptive effect of morphine. Latencies in seconds to nociceptive response in male and female GLP-1 receptor nestin-Cre KO and WT mice tested on a 55°C hot-plate after pretreatment with saline or Ex4 (10 μg/kg) and administration of (***A***) saline, (***B***) 5-mg/kg morphine, or (***C***) 10-mg/kg morphine, with a cutoff at 40 s. Data are shown as the % of mice showing a response as a function of time. Latencies did not differ significantly by genotype or Ex4 treatment for saline or 5-mg/kg morphine. For 10-mg/kg morphine, there was a genotype by Ex4 interaction and a trend for reduced latency in the Ex4-treated WT mice relative to saline but not in the KO mice; *n* = 11–14 (morphine doses) and *n* = 23–27 (saline, combined from both dose cohorts).*Figure Contributions*: Annika Billefeld Bornebusch and Gitta Wörtwein collected the data. Morgane Thomsen and Annika Billefeld Bornebusch designed the experiments and analyzed the data.

Hot-plate latencies were analyzed for each morphine dose and are shown in Figure 7. Sex was never a significant factor; therefore, data are reported as sexes combined. After administration of saline (Fig. 7*A*) or 5-mg/kg morphine (Fig. 7*B*), latency to nociceptive response was not significantly related to genotype or Ex4 condition. At 10-mg/kg morphine, there was a significant effect of Ex4 treatment (*p* = 0.045, Cox proportional hazard regression) and significant genotype by Ex4 interaction (*p* < 0.001; Fig 7*C*), while the main effect of genotype approached significance (*p* = 0.075). The interaction reflected that the Ex4 effect approached significance to reduce latency in the WT mice (*p* = 0.054) but not in the KO mice (*p* = 1.0).

## Discussion

Our findings in the present study indicate that GLP-1 receptor agonists do not reduce abuse-related effects of opioids in assays of reward, reinforcement, or withdrawal, as well as morphine-induced hyperactivity.

Morphine produces place preference in the CPP assay, which is related to the perception of opioid reward (Bardo and Bevins, 2000). GLP-1 receptor agonists have been shown to block or reduce CPP conditioned by alcohol, cocaine, amphetamine, and nicotine (Egecioglu et al., 2013a, b,c; Graham et al., 2013; Shirazi et al., 2013; Harasta et al., 2015; Vallöf et al., 2016; Sirohi et al., 2016). In contrast, we found that Ex4 pretreatment did not alter acquisition of morphine-conditioned CPP, despite using a moderately high dose (10 μg/kg) compared to 2.4-µg/kg Ex4 being sufficient to prevent acquisition and/or expression of CPP in previous studies (Egecioglu et al., 2013a, b,c; Sirohi et al., 2016). The doses used in the present investigation were based on preliminary studies showing no effect of lower doses Ex4 with morphine (data not shown), and 10 µg/kg was selected as the highest dose that would not induce side effects to a degree that would interfere with interpretation of the data. Indeed, 10-µg/kg Ex4 by itself produced significant decreases in locomotor activity during the conditioning sessions, relative to saline. This effect is consistent with previous findings (Krass et al., 2012; Sørensen et al., 2015) and confirms that the Ex4 treatment used was “active” in these investigations. A dose of 10-µg/kg Ex4 alone did not produce conditioned place aversion, indicating that aversive effects of the Ex4 treatment at the doses used in our study are unlikely to contribute significantly to the behavioral outcomes observed.

We also tested whether Ex4 treatment would decrease the reinforcing effects of an opioid drug, i.e., active drug taking behavior. Ex4 did not attenuate the reinforcing properties of remifentanil in WT mice during acquisition, extinction, rebaseline, or FR 1 dose-response testing. A smaller number of animals were also tested under increased response requirement conditions. If anything, the tendency for Ex4 to increase remifentanil self-administration under the post-extinction rebaseline and increased response requirement conditions would suggest an increased reinforcing effect of remifentanil. This is again in contrast to other addictive substances: GLP-1 receptor agonists decreased IVSA of alcohol, cocaine, and nicotine (Sørensen et al., 2015, 2016; Schmidt et al., 2016; Tuesta et al., 2017) and oral self-administration of alcohol (Egecioglu et al., 2013c; Vallöf et al., 2016). Importantly, we confirmed that 3.2- and 10-µg/kg Ex4 significantly decreased oral alcohol self-administration in mice under the same experimental conditions as our remifentanil IVSA studies.

The IVSA experiment included mice lacking GLP-1 receptors specifically in the central nervous system but expressing GLP-1 receptors in peripheral tissues. Perhaps not surprisingly in light of the lack of effect of Ex4 treatment, we found no difference in IVSA behavior between WT and KO mice. The exception was a genotype by treatment interaction during post-extinction rebaseline, suggesting that Ex4 increased remifentanil IVSA in the WT mice but not in the KO mice, consistent with an effect mediated through central GLP-1 receptors. A trend for higher responding under the PR schedule of reinforcement in the KO mice was most likely not drug-specific, because KO mice reached significantly higher breaking points than WT mice when tested with food. The constitutive lack of GLP-1 receptors may lead to an increased responsiveness to positive reinforcers generally. Indeed, similar to genetic ablation, systemic or intracranial administration of a GLP-1 receptor antagonist can increase intake of sucrose, fat, and alcohol, and increased sucrose-reinforced responding under a PR schedule in rats (Knauf et al., 2008; Shirazi et al., 2013; Terrill et al., 2016). Behavior under increased response requirement was marked by strong variability, which may be related to the mixed genetic background (129, SJL and backcrossed to C57BL/6J) or to the nestin-Cre manipulation rather than GLP-1 receptor genotype. For this reason, i.e., the possibility of confounding genetic factors, and because there was no indication that Ex4 would decrease remifentanil IVSA with higher group sizes, we decided against operating and training additional animals for testing under the PR schedule, since there was no clear benefit to counter the ethical and resource costs.

In humans, opioid drugs produce physical dependence that contributes heavily to continued drug use (Weiss et al., 2014). In rodents, opioids similarly induce dependence-like behaviors after a few exposures, as defined by withdrawal symptoms being precipitated by administration of a mu opioid receptor antagonist or by abrupt cessation of opioid treatment (Schulteis et al., 1997). We tested whether Ex4 could prevent the induction of dependence-like behavior and/or the expression of withdrawal symptoms. Neither administration of Ex4 during induction of dependence nor administration of Ex4 on the test day decreased the severity of withdrawal symptoms. In mice, morphine also produces hyperlocomotion, which was also not attenuated by Ex4, measured during the conditioning sessions of the CPP experiment, despite decreasing spontaneous (saline) locomotor activity. Taken together, GLP-1 receptor agonist treatment, acute or subchronic, was ineffective at decreasing opioid reward, reinforcement, or dependence/withdrawal, even at doses higher than those needed to significantly disrupt abuse-related behavioral and biochemical effects of alcohol, stimulants, and nicotine.

Finally, we tested whether Ex4 treatment would alter the analgesic effects of morphine. Ex4 did not produce an analgesic or hyperalgesic effect in itself in the hot-plate test. While Ex4 has shown antinociceptive effects in hypersensitivity models, our lack of effect on acute pain perception is consistent with previous reports (Möller et al., 2002; Gong et al., 2014). Ex4 treatment tended to reduce the effect of morphine in the hot-plate test in WT mice, but because most mice reached the cutoff time, making statistical power weaker, it is difficult to conclude based on those data. A possible interaction between GLP-1 agonists and morphine in analgesia tests may be worth investigating further.

Because GLP-1 receptor agonists have been shown to attenuate abuse-related effects of alcohol, central stimulants (cocaine and amphetamine), and nicotine in rats and mice, it seems surprising that Ex4 had no attenuating effect on the abuse-related effects of morphine or remifentanil in the present investigation. This apparent discrepancy is not easily explained by differences in ligand (Ex4 is the most thoroughly tested agonist with other drugs of abuse), species, strain, sex, type of assay, acute versus chronic dosing, dose range, ligand formulation, or even laboratory/experimenter. Rather, this difference likely informs the mechanism of action of GLP-1 receptor agonists on reward pathways, which are still poorly understood. Studies using direct infusion of GLP-1 receptor agonists into the brain, KO mice lacking GLP-1 receptors in specific tissues, or chemogenetic stimulation of specific cell populations converge to strongly indicate that reduced intake of alcohol, central stimulants, and nicotine depend on GLP-1 receptor stimulation in the brain, rather than in peripheral tissues (Shirazi et al., 2013; Harasta et al., 2015; Sirohi et al., 2016; Tuesta et al., 2017; Hernandez et al., 2019). These studies have pointed to brain areas known to be important for reward/addiction and that express GLP-1 receptors at low to moderate levels (NAc, VTA), as well as to areas enriched in GLP-1 receptors and whose roles in addiction or reward and recently being (re)discovered (lateral septum, interpeduncular nucleus) as possible targets for the GLP-1 analogs (Shirazi et al., 2013; Harasta et al., 2015; Reddy et al., 2016; Schmidt et al., 2016; Tuesta et al., 2017; Abtahi et al., 2018; Hernandez et al., 2018, 2019). How the apparent effects of GLP-1 receptor stimulation in those various brain regions are related at a circuit level and how the effects are mediated at the neuronal or molecular level remains to be elucidated. However, no obvious drug class separation emerges from this picture, since VTA, NAc, and lateral septum have also been implicated in the addiction-related effects of opioids (Le Merrer et al., 2007; Fields and Margolis, 2015; Jiang et al., 2018; but see Perrotti et al., 2008 on differential ΔFosB expression in lateral septum).

In particular, disinhibition/stimulation of dopaminergic neurons in the relatively heterogeneous region commonly referred to as the VTA are thought to play a critical role in the reinforcing effects of alcohol, nicotine, and opioids. Recent evidence indicates that stimulation of GLP-1 receptors in the VTA weakens synaptic strength of VTA-NAc projections in mice (Wang et al., 2015b), which is probably downstream of the effects of alcohol, nicotine, and opioids on VTA neurons. Studies in rats indicated that GLP-1 receptor stimulation in the VTA could increase dopaminergic neuron activity via a presynaptic mechanism (Mietlicki-Baase et al., 2013). Apart from species and methodological differences between the two studies, this apparent discrepancy illustrates the complexity of GLP-1 receptor actions in the central nervous system at the synaptic level, which include both inhibitory and stimulatory effects, sometimes within the same structure (for review, see Liu and Pang, 2016). Relatively few VTA neurons express GLP-1 receptors (Cork et al., 2015; Harasta et al., 2015), and rewarding and aversive stimuli including opioids and other drugs may inhibit and stimulate different populations of dopaminergic VTA neurons (Lammel et al., 2011, 2012; Fields and Margolis, 2015). It is therefore possible that GLP-1 receptors are only expressed on neuron populations that are modulated preferentially by some drugs. To understand how GLP-1 systems influence reward and addiction, it will be important to better identify the neuron populations that express GLP-1 receptors, and the synaptic and cellular effects of GLP-1 receptor stimulation in specific pathways.

At the behavioral level, GLP-1 receptor stimulation may reduce appetitive effects of alcohol, central stimulants, and nicotine, or may enhance effects that limit intake, such as aversion and/or satiety, or both. For instance, Tuesta et al., (2017) suggested that stimulation of GLP-1 receptors in the medial habenula-interpeduncular nucleus circuit decreases nicotine intake by modulating nicotine satiety. Similarly, self-administered cocaine was suggested to produce a GLP-1-dependent negative-feedback loop through activation of stress circuits including corticosterone, limiting cocaine intake (Schmidt et al., 2016), which could also be interpreted as a form of GLP-1-potentiated cocaine “satiety.” Such mechanisms would be in line with the well-established role of GLP-1 circuits to regulate nutrient intake. In this respect, it is unclear how opioids may differ from other drug classes in “satiating” or aversive effects. Some authors argue that, at least in the acquisition of drug taking stage, opioids produce an almost pure euphoric (approach) response in humans and rodents, while cocaine produce mixed euphoric/aversive (approach/avoidance) effects (Badiani et al., 2011). However, this difference between cocaine and heroin in approach/avoidance behavior is most likely pharmacokinetic, with the shorter-acting cocaine producing euphoric and aversive (“crash”) effects in closer temporal proximity relative to heroin, D-amphetamine, or methamphetamine (Ettenberg, 1990, 2004, 2009; Akhiary et al., 2018). Mixed appetitive/aversive effects can be observed for all major classes of abused drugs including opioids, in humans and laboratory animals (Angst et al., 2012; Verendeev and Riley, 2013). Nevertheless, is it possible that GLP-1 receptor stimulation decreases drug taking and drug seeking by potentiating some aversive or satiating effects of alcohol, stimulants, and nicotine that are not shared by opioids. For instance, rewarding versus aversive effects of opioids may be more distinctly dependent on central versus peripheral mechanisms (Bechara and van der Kooy, 1985). It would be useful to better understand the nature of GLP-1-mediated modulation of drug taking and alcohol taking and seeking behaviors, in terms of satiety, motivation, craving, direct reinforcing and aversive/punishing strength, and salience of conditioned reinforcers/punishers associated with drug/alcohol taking.

Regarding the possible involvement of stress systems (cf. Schmidt et al., 2016), corticotropin-releasing factor (CRF)-releasing neurons in the hypothalamus express GLP-1 receptors, and GLP-1 receptor stimulation increases CRF signaling (Larsen et al., 1997; Kinzig et al., 2003; Ghosal et al., 2017; Liu et al., 2017). However, at the systemic level, activation of stress systems likely contributes to addictive effects or drugs more than it opposes them, at least in the later stages of addiction (for review, see Koob, 2008; Koob and Volkow, 2016), making it difficult to speculate on the role of GLP-1-mediated CRF release in “anti-addictive” effects. Moreover, GLP-1-mediated increases in CRF or corticotropin levels do not offer an obvious explanation for why GLP-1 receptor agonists attenuate effects of alcohol, stimulants, and nicotine, but not opioids. Indeed, activation of stress systems, including increased levels of CRF, are observed with chronic use of alcohol, stimulants, nicotine, and opioids, and CRF antagonists can reduce effects of chronic opioid exposure including drug seeking and withdrawal symptoms, similar to effects observed with other drug classes (Shaham et al., 1998; Stinus et al., 2005; Park et al., 2015; for review, see Koob, 2008). While most studies have measured acute effects of GLP-1 receptor agonists, GLP-1 agonist treatment can also attenuate effects of alcohol in more chronic experimental designs, when stress systems are presumably in play. GLP-1 receptor agonists appeared to attenuate ethanol withdrawal symptoms and craving, based on measures of anxiety-like behavior and alcohol deprivation-induced drinking (Sharma et al., 2015; Vallöf et al., 2016; Thomsen et al., 2017). GLP-1 agonists reduced alcohol intake in non-human primates with many months history of drinking (Thomsen et al., 2018), and one GLP-1 analog was effective in decreasing drinking only in mice with repeated exposure to ethanol and presumed to have developed ethanol dependence (Suchankova et al., 2015). Taken together, activation of stress systems does not seem a likely general mechanism by which GLP-1 receptor agonists decrease drug seeking and drug taking.

We hypothesize that GLP-1 receptor agonists may effectively attenuate abuse-related effects that are specifically dependent on striatal dopamine D1 receptor (Drd1) pathway signaling. There is strong evidence that alcohol, central stimulants, and nicotine all produce addictive effects dependent on Drd1-dependent mechanisms. Reinforcing and rewarding effects of alcohol, cocaine, and nicotine can be attenuated by blocking/decreasing Drd1 pharmacologically or genetically, and blockade in the nucleus accumbens shell appears sufficient to produce at least some of those effects (Corrigall and Coen, 1991; David et al., 2006; Bahi and Dreyer, 2012; Young et al., 2014; Ding et al., 2015; Pisanu et al., 2015). The same manipulations did not impair the reinforcing effects of heroin in a direct comparison (Pisanu et al., 2015). Studies using KO mice further support differences between alcohol or stimulants versus opioids. KO mice lacking Drd1 receptors showed no reinforcing effects of cocaine and strongly reduced alcohol drinking, but showed normal remifentanil self-administration and normal morphine CPP (El-Ghundi et al., 1998; Caine et al., 2007; Urs et al., 2011; Ting-A-Kee et al., 2013). Conversely, KO mice lacking Drd2 receptors failed to self-administer morphine and failed to develop morphine CPP or morphine withdrawal-conditioned aversion, but self-administered cocaine to overdose and showed normal cocaine CPP (Caine et al., 2002; Elmer et al., 2002; Smith et al., 2002). Opioids and other drug classes also produce qualitatively different adaptations of Drd1-expressing and Drd2-expressing medium spiny neurons (MSNs) at the molecular level. Cocaine, alcohol, and tetrahydrocannabinol all induce ΔFosB specifically in Drd1-expressing MSNs, whereas morphine and heroin induce ΔFosB in both Drd1 and Drd2-expressing MSNs (Kelz et al., 1999; Lobo et al., 2013; see also Wang et al., 2015a). Acute effects of opioids may involve Drd1-dependent mechanism, but after chronic exposure and cycles of dependence and withdrawal, the mechanism appears to switch to more Drd2-dependent mechanisms (Georges et al., 2000; Lintas et al., 2011; Enoksson et al., 2012). In contrast, cocaine affects both Drd1-and Drd2-expressing neurons acutely, albeit in different ways (Bertran-Gonzalez et al., 2008; Luo et al., 2011), but repeated administration appears to shift effects toward Drd1-expressing neurons almost exclusively (Lee et al., 2006; Bertran-Gonzalez et al., 2008). For instance, cocaine-induced increases in dendritic spine density occurs in Drd1 and Drd2 MSNs, but is maintained long-term only in Drd1 MSNs (Lee et al., 2006). Thus cocaine and morphine may produce addictive effects by producing similar shifts in the balance between Drd1 and Drd2 MSNs activity but through different mechanisms, one modulating Drd1 neurons, the other, Drd2 neurons (Koo et al., 2014; Graziane et al., 2016; Renteria et al., 2017). It is not known whether Drd1/Drd2 subpopulations of MSNs are affected differentially by GLP-1 manipulations, but recent studies indicate that Ex4 can modulate MSNs excitability in cocaine-experienced but not drug-naive rats, and that this response was observed only in roughly half the recorded MSNs (Hernandez et al., 2019).

The shift from acute drug effects to addiction and/or dependence is accompanied by neuroplasticity changes in the brain. While many such changes are observed across drug classes, such as altered glutamatergic transmission and redistribution of AMPA receptors (Saal et al., 2003; Brown et al., 2010), there also are important differences. For instance, self-administration of opioids but not cocaine, alcohol, or nicotine was observed to cause morphologic changes in dopaminergic VTA neurons (Mazei-Robison et al., 2014). Chronic self-administration of and/or passive exposure to cocaine, amphetamine, or nicotine increases dendritic spine density in NAc MSNs and in prefrontal cortex, while morphine exposure under comparable experimental conditions *decreases* dendritic spine density, and alcohol produces more complex alterations in spine morphology (Brown and Kolb, 2001; Robinson et al., 2001, 2002; Zhou et al., 2007; Uys et al., 2016; for review, see Robinson and Kolb, 2004; Russo et al., 2010). It is unclear whether such differences in the maladaptive changes to chronic drug exposure can explain the lack of effect of Ex4 on acute effects of opioids (as well as repeated-exposure effects) in the present study, although neuroplasticity changes may begin to form after one or a few drug exposures. In elucidating how GLP-1 receptor stimulation modulates addictive effects of alcohol or drugs, it would be informative to examine the effects of GLP-1 analog treatments on neuroplasticity changes such as spine/neuron morphology and glutamate receptor characteristics.

In conclusion, unlike other addictive substances investigated so far, Ex4 did not attenuate the addiction-related behavioral effects of opioids in mice, and we found no indication that GLP-1 analogs would have a beneficial effect in the medical treatment of opioid dependence. This difference between opioids and other drug classes may shed light on the mechanism of action of GLP-1 receptor treatment in the addictive effects of alcohol, central stimulants, and nicotine.

## Synthesis

Reviewing Editor: Christian Lüscher, University of Geneva Decisions are customarily a result of the Reviewing Editor and the peer reviewers coming together and discussing their recommendations until a consensus is reached. When revisions are invited, a fact-based synthesis statement explaining their decision and outlining what is needed to prepare a revision will be listed below. Note: If this manuscript was transferred from JNeurosci and a decision was made to accept the manuscript without peer review, a brief statement to this effect will instead be what is listed below.

1. the authors refer to oral alcohol self-administration (ASA) as a positive control. I wonder if that is correct. The ASA is an important experiment and my comment is perhaps just semantic. But it seems misleading as the authors tested if GLP-1 was effective on ASA (but they did not know a priori that it was going to work on ASA, even if previous literature would have suggested so).

2. Some literature is missing, including a 2015 human genetic and preclinical work with a GLP-1 analog (Suchankova P, et al. Translational Psychiatry)

3. It would be interested to test the effects, if any, of a GLP-1 antagonist (e.g. exendin-(9-39): Thorens B, Porret A, Buhler L, Deng SP, Morel P, et al. Diabetes 1993) on these opioid-related experiments and outcomes. At minimum, I would test remifentanil given the authors' findings that, if anything reinforcing effects of remifentanil appeared increased in Ex-4 treated mice relative to saline. That is, would GLP-1 antagonism lead to decrease reinforcing effects of remifentanil?

4. I would encourage the authors to expand the discussion on the potential reasons why EX-4 seems to be selective for some drugs of abuse (e.g. alcohol) and not for others. I do appreciate that the authors do not want to make the Discussion too speculative, but a few more thoughts could help the reader understanding e.g. which next directions could be taken.

## Author Response

Glucagon-Like Peptide-1 receptor agonist treatment does not reduce abuse-related effects of opioid drugs

We thank the editor and reviewers for thoughtful comments. Revisions in response to the comments are highlighted in yellow in the revised manuscript. Point-by-point responses and changes made:

1. In the introduction, we have reworded the “positive controls” as: “we verified the effectiveness of Ex4 using endpoints previously reported to be sensitive to GLP-1 receptor stimulation, namely locomotor activity and oral alcohol self-administration, measured in direct comparison to the opioid assays.” (page 6). In the remainder of the manuscript, we have replaced “ethanol self-administration control experiment” with “ethanol self-administration experiment”.

2. Several citations have been added, including Suchankova et al. 2015 (p.5 and 6), the human studies van Bloemendaal et al. 2014 and Kalra et al. 2011 (p.5), the preclinical studies Graham et al. 2013 and Shirazi et al. 2013 (p. 6 and 9), and the clinical trial protocol Yammine et al. 2018 (p. 6).

3. We agree that it would very interesting to test a GLP-1 antagonist. Unfortunately, we find ourselves unable to add the requested experiments within the next months, as we are moving to newly constructed laboratory facilities, with all the delays and problems this always seems to entail. We did test effects of the GLP-1 antagonist exendin-(9-39) in expression of morphine withdrawal. There was no indication that Ex9 decreased withdrawal signs, but the level of behavior in the control group was significantly lower than the previous control groups (several ‘zero’ jumps in both groups, although a huddled posture etc. suggests withdrawal was induced). Therefore, we feel we would need to repeat the experiment to insure the data is of publishable quality. The test was not done under optimal conditions as the old facilities were being closed down: renovation noises, disinfectant smells, or activity of personnel packing up equipment in adjacent rooms may have disturbed the animals and interfered with ‘normal’ behavior. Self-administration behavior is even more susceptible to disturbances and was not attempted.

4. We have expanded the discussion section substantially as suggested.
